# Population Structure and Genomic Characteristics of Australian *Erysipelothrix rhusiopathiae* Reveals Unobserved Diversity in the Australian Pig Industry

**DOI:** 10.3390/microorganisms11020297

**Published:** 2023-01-23

**Authors:** John Webster, Bethany Bowring, Leah Stroud, Ian Marsh, Narelle Sales, Daniel Bogema

**Affiliations:** 1 NSW Department of Primary Industries, Elizabeth Macarthur Agricultural Institute PMB 4008, Narellan, NSW 2570, Australia; 2 Centre for Infectious Diseases and Microbiology, The Westmead Institute for Medical Research, Westmead, NSW 2145, Australia; 3 Department of Microbiology and Infectious Diseases, Sydney South West Pathology Service, Liverpool, NSW 2170, Australia

**Keywords:** *Erysipelothrix*, pig, population, genomics

## Abstract

*Erysipelothrix rhusiopathiae* is a bacterial pathogen that is the causative agent of erysipelas in a variety of animals, including swine, emus, turkeys, muskox, caribou, moose, and humans. This study aims to investigate the population structure and genomic features of Australian isolates of *E. rhusiopathiae* in the Australian pig industry and compare them to the broader scope of isolates worldwide. A total of 178 isolates (154 Australian, seven vaccine isolates, six international isolates, and 11 of unknown origin) in this study were screened against an MLST scheme and publicly available reference isolates, identifying 59 new alleles, with isolates separating into two main single locus variant groups. Investigation with BLASTn revealed the presence of the *spaA* gene in 171 (96%) of the isolates, with three main groups of SpaA protein sequences observed amongst the isolates. Novel SpaA protein sequences, categorised here as group 3 sequences, consisted of two sequence types forming separate clades to groups 1 and 2, with amino acid variants at positions 195 (D/A), 303 (G/E) and 323(P/L). In addition to the newly identified groups, five new variant positions were identified, 124 (S/N), 307 (Q/R), 323 (P/L), 379 (M/I), and 400 (V/I). Resistance screening identified genes related to lincomycin, streptomycin, erythromycin, and tetracycline resistance. Of the 29 isolates carrying these resistance genes, 82% belonged to SpaA group 2-N101S (*n* = 22) or 2-N101S-I257L (*n* = 2). In addition, 79% (*n* = 23) of these 29 isolates belonged to MLST group ST 5. Our results illustrate that Australia appears to have a unique diversity of *E. rhusiopathiae* isolates in pig production industries within the wider global context of isolates.

## 1. Introduction

*Erysipelothrix rhusiopathiae* is a Gram-positive, rod-shaped bacterium that is the causative agent of erysipelas in a variety of animals, such as birds and mammals, including swine, emus, turkeys, muskox, caribou, moose, and humans [1,2,3]. *E. rhusiopathiae* is a facultative intracellular pathogen that is best known for causing erysipelas in all stages of pig production [4,5], clinically presenting as acute, subacute, and chronic forms of the disease. In the acute form of the disease, pigs may die suddenly without showing clinical symptoms, and sub-acutely affected pigs may present with a fever and walking stiffly on their toes and developing characteristic diamond shaped skin lesions, while chronic forms of the disease result in arthritis and heart problems [6]. As such, *E. rhusiopathiae* is important economically in pigs, as outbreaks can cause major losses that are associated with high morbidity and mortality rates during an outbreak and result in animal loss and carcass condemnation [4,6]. Vaccine usage is routine among pig breeding herds in Europe, North America, and South America and control of erysipelas in pigs was successful for decades [4,6,7]. However, recent outbreaks such as in the US, Japan, the UK, and China suggest that there is now a re-emergence of swine erysipelas, as well as in the European poultry industry [8,9,10,11,12]. In 2012, *E. rhusiopathiae* was attributed to a largescale mortality event of muskoxen (*Ovibos moschatus wardi*) on Banks Island in the Northwest Territories, Canada [13], and prior to then, had only been observed in four dead wood bison near Fort Smith 40 years earlier [14]. The mortality events of 2012 led to a review and testing of archived samples from other mortality events through 2009–2011, which found further evidence of *E. rhusiopathiae* infections [13].

Vaccination of swine for erysipelas is regularly performed worldwide and has been shown to positively affect sow fertility and birth rates as well as perform a preventative role in the acute and peracute forms of the disease [15,16,17]. Historically, attenuated vaccines were originally used to confer protection against erysipelas and moved in the 1940s to inactivated bacterial components consisting of a soluble immunogenic protein, later discovered to be the surface protein antigen (spa) [18,19]. Previous studies of *E. rhusiopathiae* have investigated the presence and sequence structure of the surface protein antigen [4,20,21]. The Spa protein has three variants, SpaA, SpaB, and SpaC, with SpaA highly conserved within *E. rhusiopathiae*. This SpaA protein is responsible for protecting pigs against erysipelas and is a major virulence determinant [19,22,23]. Novel vaccine approaches currently also focus on SpaA’s immunogenic properties, with many modern vaccines utilising some form of SpaA to induce protection [6].

This study aims to investigate the diversity of Australian *E. rhusiopathiae* isolates obtained over the last five decades and compare them to the broader scope of isolates worldwide.

## 2. Materials and Methods

### 2.1. Antibiotic Resistance Profiling

Antibiotic susceptibility profiling was performed using a modified version of the calibrated dichotomous susceptibility (CDS) protocol for disc diffusion. Three to six colonies of *E. rhusiopathiae* isolates were suspended in saline and flooded onto the surface of Sensitest blood agar plates (MicroMedia, Sydney, Australia). After 15 min, any excess inoculum was removed, and six antibiotic disks (Oxoid, Scoresby, Australia) (Supplementary Table S1) were dispensed onto the plate. Plates were incubated at 35 °C with 10% carbon dioxide for 48 h. *Staphylococcus aureus* (NTC6571) was included as a positive control throughout the testing. The results from the testing were reported as either susceptible (≥6 mm) or resistant (<6 mm) based on the zone of inhibition as specified in the CDS protocol by Bell et al., 2016 [24].

### 2.2. Extraction and Purification of DNA for WGS

DNA was extracted using the QuickGene DNA Tissue kit (Kurabo, Osaka, Japan) in combination with the QucikGen-810 (Kurabo, Osaka, Japan). After extraction, the DNA was subjected to the following quality control analyses before submission for whole genome sequencing (WGS); the DNA had to be intact double-stranded DNA, as assessed by agarose gel electrophoresis; the DNA concentration required for NextEra XT sequencing was measured using the Qubit Fluorimeter and a Nano Drop spectrophotometer measured the 260/280 ratio to check for RNA contamination. DNA was confirmed as being intact by agarose gel electrophoresis, and it was measured for concentration by Qubit Fluorimeter, as well as purity by a Nano Drop spectrophotomer. Samples were submitted to the Ramaciotti Centre for Genomics (Sydney, Australia) where NextEra XT WGS was undertaken.

### 2.3. Genome Assembly and Annotation

WGS reads were assembled using Spades assembler (v.3.11.1) [25]. Raw sequencing data was quality checked for accuracy and contamination using fastqc (https://www.bioinformatics.babraham.ac.uk/projects/fastqc/ accessed on 25 November 2019) and kraken [26]. Assembled genome sequences were checked using QUAST (v.4.6.3) [27] and BlobTools (v.1.0) [28] to assess genome completeness, fragmentation, and to further exclude contaminating DNA sequences. These steps were automated using asqcan (https://github.com/bogemad/asqcan, accessed on 15 December 2022). *E. rhusiopathiae* short read data from the NCBI short read archive (SRA), which was downloaded and assembled also using asqcan.

### 2.4. Identification of SpaA, Surface Proteins and Antimicrobial Resistance Genes

All genomes were screened for *spa* genes using BLASTn v.2.10.1 and a reference list of *spaA*, *spaB*, and *spaC* sequences presented in Forde et al., 2020 [4]. Resulting spa sequences from isolates in this study were translated and then aligned using Clustal Omega 1.2.2 [29]. The hypervariable region between amino acid residues 30 and 413 was screened in Geneious Prime (Build 23 July 2020 08:02) for amino acid variants. Surface proteins were also screened in all genomes using Ariba v.2.14.6 [30]. Genomes were initially shredded with randomreads.sh [31] to produce simulated short reads that were subsequently screened with Ariba to identify genes for the following proteins: rhusiopathiae surface protein A (*rspA*), choline-binding protein (*cbpB*), type I glyceraldehyde-3-phosphate dehydrogenase (GAPDH), dipeptidyl aminopeptidase (*dap2*), collagen-binding protein (*cbpA*), pectin lyase fold-containing protein (Plp), ABC transporter (*Atsp*), neuraminidase (*nanH*), beta-galactosidase (*Bga*), basic membrane lipoprotein (*Bml*), and LPXTG-motif cell wall anchor domain proteins (*cwpA*/*cwpB*). Ariba outputs were then parsed with a custom in-house script to identify whether variants in the isolates compared to the reference (Fujisawa strain NC_015601) were either (i) completely synonymous, (ii) contained a non-synonymous mutation, or (iii) contained a frameshift/indel.

Genomes were also screened for resistance genes against 3077 nucleotide sequences in the ResFinder database (version as of 6 April 2021) [32] using Abricate v.1.0.1 [33].

### 2.5. Multi Locus Sequence Typing

Genomes sequenced in this study and assembled from the SRA database, were screened against the Janßen et al. [20] MLST scheme using mlst v.2.19.0 [34] with novel alleles output using the ‘-novel’ flag. Novel alleles were assigned new identifiers, and the MLST scheme was updated to incorporate all novel alleles. Lastly, mlst was run with all isolates against the updated MLST scheme to determine sequence types (ST) for each isolate. A minimum spanning tree (MST) was then computed in PHYLOViZ Online [35] with the goeBURST full MST function.

### 2.6. Phylogenetic Inference

A core gene phylogeny of reference and sequenced *E. rhusiopathiae* isolates was performed using Core_gene_phylo [36]. Panaroo v.1.2.9 [37] was employed to calculate the set of genes core to all isolates. These genes were then model tested with IQTree v.2.1.4-beta [38] and those that passed testing retained and a supermatrix constructed. ClonalFrameML v.1.12 [39] was used for recombination filtering, and a maximum likelihood tree was inferred with a general-time reversible model and a discrete-gamma substitute rate, with 1000 bootstrap replicates performed. The bootstrap replicate tree was finally rooted using minimum-variance rooting with FastRoot.py v.1.5 [40].

### 2.7. Estimating Recombination Rates

To determine the recombination and mutation event rates (μ and γ respectively) of *E. rhusiopathiae* phylogenetic clades, mcorr v.20180102 [41] was run on core gene alignments produced from Panaroo, with 1000 bootstrap replicates. Mcorr uses a coalescent-based model of evolution to measure the degree that any two loci l bp apart have correlated substitutions and estimates six evolutionary parameters: dsample—the amount of diversity brought into the sample by recombination, θ—the average number of mutations per locus, ϕ—the mean number of recombinations per locus, θ/ϕ—the rate of recombinations to mutations (equal to γ/μ), $\bar{f}$—the average fragment size of a recombination event, and c—the recombination coverage of the sample (i.e., proportion of sites whose diversity has come from outside the sample). Welch’s *t*-tests were then used to compare mcorr parameters between clades.

## 3. Results

### 3.1. Population Structure of E. rhusiopathiae

**Figure 1:**
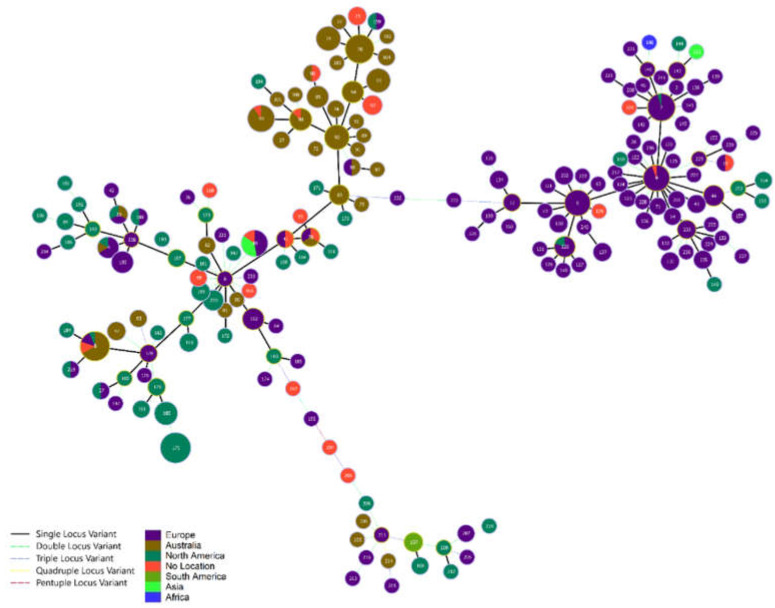
Minimum spanning tree of *E. rhusiopathiae* MLST scheme created from sequence of genes *pta*, *galK*, *purA*, *ldhA*, *recA*, *gpsA*, and *prsA*. Two major single locus variants (SLVs) consisting of a European group of isolates and a second SLV of isolates from Australia and the Americas.

**Figure 2:**
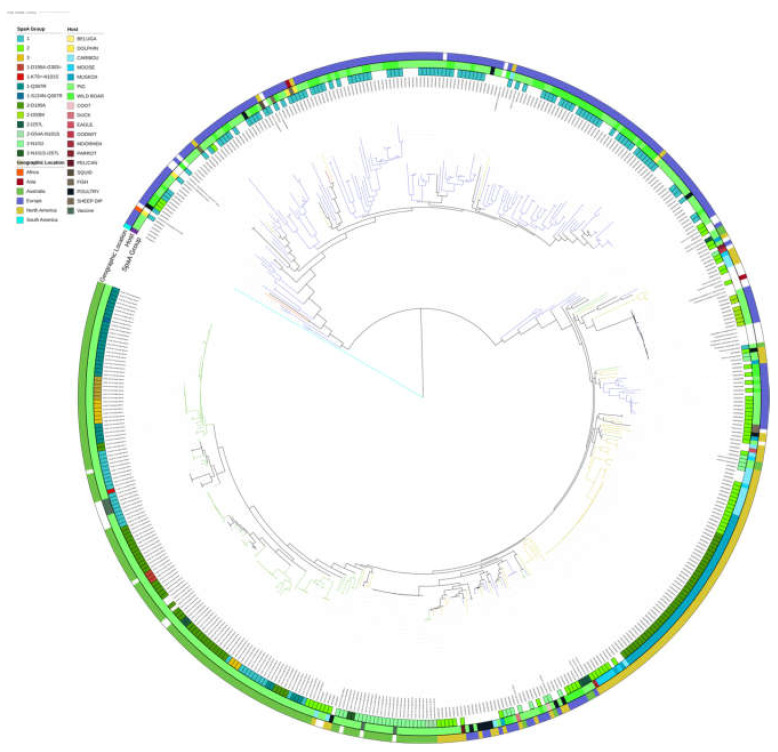
Core gene phylogenetic tree inferred with core_gene_phylo from 511 genes. Annotated rings, moving inside to out, show: SpaA protein group identified in the isolate, presence of surface proteins, host the sample was isolated from, and geographic location of isolation. Branches are also coloured to reflect the geographic location.

Screening of the sequenced isolates in this study, as well as SRA isolates, revealed 59 novel alleles within the *Erysipelothrix* MLST scheme. Of these, 13 novel alleles were found for *pta*, 10 for *galK*, six for *purA*, nine for *ldhA*, 11 for *recA*, six for *gpsA*, and four for *prsA*. In addition, 122 new ST were identified within the investigated genomes. In total, 170 ST were assigned to *E. rhusiopathiae* isolates, and of those, 107 (63%) were singletons. The most common ST, ST175, was represented by 40 (8.4%) isolates, with the top 15 ST representing 50% of the screened isolates. A total of 37 STs were assigned to samples from genome sequenced Australian isolates. Two large single locus variant (SLV) groups were observed within the MLST (Figure 1) forming a SLV group of predominantly European isolates and a second group of Australian and American isolates. This separation was also observed in the core gene phylogenetic tree (Figure 2) with two major clades, one representing the European isolates and a second representing a mixture of American and Australian isolates, with the majority of Australian isolates forming their own subclade. Phylogenetic inference of 506 *E. rhusiopathiae* isolates from 511 core genes identified with Panaroo was also performed. Australian isolates were observed to cluster primarily within the same clade as the American isolates, previously identified as ‘Clade 3’ by Forde et al. (2020). Phylogenetic inference also identified ‘Clade 2’ to be composed of isolates of European sample origin and the ‘intermediate clade’ reported by Forde et al. (2020). Sequence types of SpaA were observed to correlate with Clades 2 and 3; Clade 2 was comprised solely of SpaA group 1 sequences, while Clade 3 contained all SpaA group 2 sequences, except for four isolates: ERR3932985, ERR3932944, ERR3932958, and ERR3932942. This correlation of SpaA sequence types also confirms the previously reported SpaA population structure observed by Forde et al. (2020).

### 3.2. Presence of SpaA Proteins in E. rhusiopathiae

**Figure 3:**
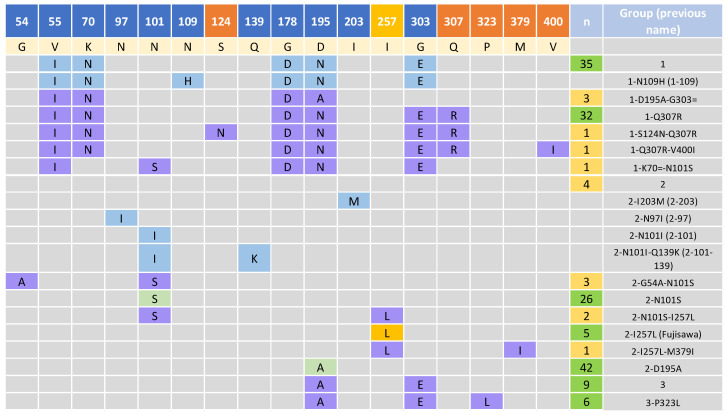
Adapted figure from Forde et al. showing discriminatory amino acid positions within the SpaA protein’s hypervariable region of *E. rhusiopathiae*. Variant positions and amino acids are relative to the Fujisawa reference sequence and are shown in the first and second rows, respectively (except position 257). Positions in orange represent novel positions observed in this study, while position 257 represents a variant position only previously observed as a variant in the Fujisawa reference genome (L). Previously reported groups and variant positions by Forde et al. are shown in blue, the variants 2-D195A and 2-N101S previously identified by Uchiyama et al., 2016 [42] and Janßen et al., 2015 in light green. Novel groups and their variants are shown in purple. The number of isolates from this study are shown for each group, in green for groups with >5 isolates and yellow for groups with <5 isolates.

**Figure 4:**
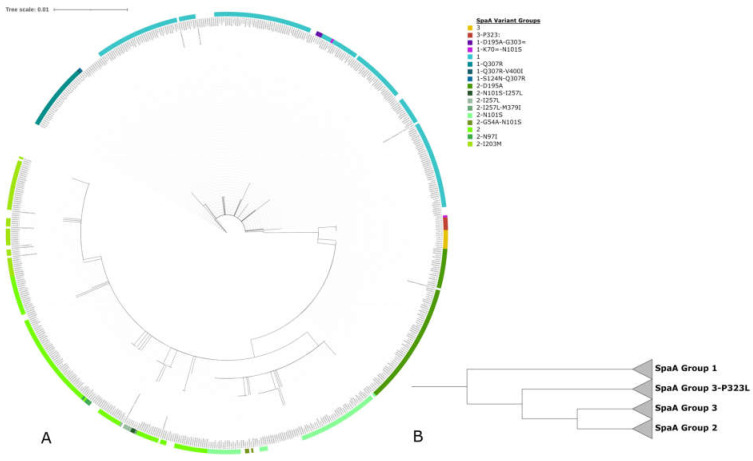
(**A**) RAxML maximum likelihood phylogeny of SpaA protein sequences over the hypervariable region from AA residues 30-413. (**B**) Collapsed branches ignoring branch length of the same SpaA tree showing the divergence of the groups 1, 2, 3 and 3-P323L.

Of 178 isolates in this study (154 Australian isolates, seven vaccine isolate, six international isolates, and 11 of unknown origin), BLASTn revealed the presence of *spaA* in 171 (96%) of the isolates. Examination of the translated hypervariable region between amino acids 30 and 413 of the SpaA proteins from the isolates revealed the presence of 15 SpaA sequence types (Figure 3). Previously reported sequence types were amalgamated by Forde et al. into two groups, designated as groups 1 and 2. In addition, two novel sequence variants are defined here as SpaA group 3. These variants were observed in 15 isolates that formed a separate clade (Figure 4) to groups 1 and 2, with amino acid variants at positions 195 (D/A) and 303 (G/E) for group 3, and a variant at position 323 (P/L) was labelled as group 3-P323L. Of the total 15 SpaA variant groups, 10 of these were novel to the dataset. Within the newly identified variant groups, three of those (1-Q307R, 3 and 3-P323L) consisted of five or more sequences and comprised over a quarter of the investigated isolates (*n* = 47, 27.5%) with an identified SpaA protein. The remaining seven novel groups (1-K70 = -N101S, 1-S124N-Q307R, 1-Q307R-V400I, 1-D195A-G303=, 2-I257L-M379I, 2-N101S-I257L, and 2-G54A-N101S) consisted of only one to three isolates (Figure 3). In addition to the newly identified groups, five new variant positions were identified, 124 (S/N), 307 (Q/R), 323 (P/L), 379 (M/I) and 400 (V/I) with variants at position 307 and 323 appearing in 34 (20%) and 6 (3.4%) isolates, respectively. A variant at position 307 had previously been reported by Forde et al., though only a single isolate was identified with a variant of Q/K, unlike the Q/R variant seen in the isolates in this study. Three isolates showed a variant in position 54, which was also observed in four isolates in the study by Forde et al. A variant at position 101 was also identified in a single isolate in Forde et al. However, the variant was N/I, as opposed to N/S observed here. Another eight isolates also included a variant at position 257 (I/L), which to date has only been reported in the Fujisawa reference genome (NC_015601.1). In total, 59/154 (39.6%) Australian isolates in this study displayed a novel SpaA sequence. In addition, this dataset (Supplementary Table S1) included two isolates from Hungary (erysip_1, erysip_42), which were identified as SpaA types 1 and 2-I257L, an isolate from Germany (erysip_53) with a SpaA type 1, an isolate from the USA (erysip_44) with a SpaA type 2-N101S, and a second isolate from the USA with no SpaA, as well as an isolate from Argentina (erysip_7) without an identified *spaA* gene.

### 3.3. Recombination Rates in E. rhusipathiae Clade 2, 3 and Australian Clades

**Figure 5:**
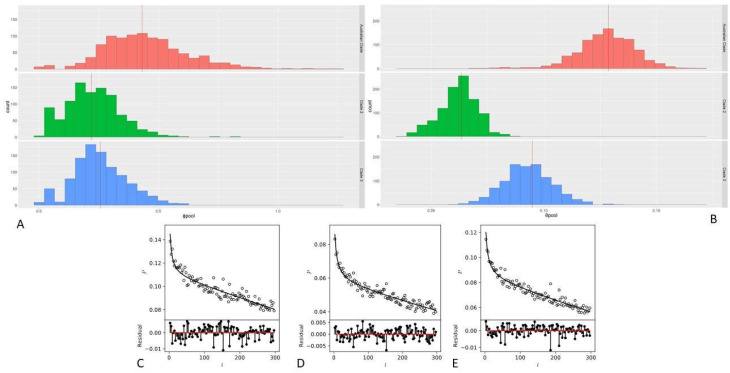
Recombination and mutation mcorr profiles. (**A**,**B**) Histograms of ϕ*_pool_* and θ*_pool_* values from 1000 bootstrap replicates of Australian clade (red), Clade 2 (green) and Clade 3 (blue) isolates. Mean values are represented by vertical red lines. (**C**–**E**) Correlation profiles analysis of (left to right) Australian Isolates, Clade 2 isolates and Clade 3 isolates of *E. rhusiopathiae*. Model fits are shown as solid lines and correlation profiles of all pairs are shown as open circles.

Recombination rates in *E. rhusiopathiae* were investigated using mcorr from Panaroo core-gene alignments. Three separate clades were investigated based on the core-gene phylogeny and previously reported clades 2 and 3 [1,4]: (1) European isolates of clade 2; (2) the Australian grouping of isolates within clade 3; (3) clade 3 isolates excluding the sub-clade of Australian isolates. Recombinational divergence was observed to be higher in the clade containing Australian isolates based on the ϕpool results of mcorr. In the Australian clade, over 1000 bootstrap replicates had a mean ϕpool value of 0.431 compared to mean ϕpool values of 0.219 and 0.256 for clades 2 and 3, respectively (Figure 5A), each displaying significantly (*p* < 0.001) different recombinational divergence profiles from each other using Welch’s *t*-tests. Mutational divergence, θpool, was also observed to be twice as high in Australian isolates (0.129) in comparison to Clades 2 (0.063) and 3 (0.095) with all groupings displaying significantly different (*p* < 0.001) mutational divergence profiles with Welch’s *t*-tests (Figure 5B). Monotonic decay of correlation profiles as a function of loci l bp apart also suggest the presence of recombination (Figure 5C–E), whereas in the absence of recombination the value of P(l) would be constant.

### 3.4. Vaccine Breakdown amongst Spa Groups

Vaccine breakdown was observed in 49 of the 178 cases examined in this study (27.5%). Of the isolated *E. rhusiopathiae* from these cases, 18 belonged to SpaA group 1 and its subgroups, 20 from SpaA group 2 and its subgroups, and 11 from SpaA group 3 and 3-P323L. This related to a total breakdown percentage of 24.7%, 24.1% and 73% for all isolates in groups 1, 2, and 3, respectively. Table 1 shows vaccine breakdown at group variant resolution. No vaccine breakdowns were observed in the six overseas isolates.

### 3.5. Surface Protein Screening

Genomes were screened for 12 additional surface proteins that have been experimentally shown to be involved in host immune responses by Forde et al. [4]. These proteins included rhusiopathiae surface protein A (*rspA*), choline-binding protein (*cbpB*), type I glyceraldehyde-3-phosphate dehydrogenase (GAPDH), dipeptidyl aminopeptidase (*dap2*), collagen-binding protein (*cbpA*), pectin lyase fold-containing protein (*Plp*), ABC transporter (*Atsp*), nueraminidase (*nanH*), beta-galactosidase (*Bga*), basic membrane lipoprotein (*Bml*), and LPXTG-motif cell wall anchor domain proteins (*cwpA*/*cwpB*).

Of the 178 genomes investigated, 95% of isolates contained all 12 surface proteins. All four isolates identified with a SpaB sequence (erysip 121, erysip 16, erysip 6 and erysip 7), were missing genes cwpB and cbpB, with a further three SpaA isolates (erysip 141, erysip 44 and erysip 66), also missing cwpB. Amongst isolates in the Australian clade, the protein sequences for dap2 were synonymous with the reference Fujisawa sequence in all isolates except erysip_106, erysip_79, erysip_10 and erysip_11, while isolates not within the Australian clade or intermediate clade majorly presented with non-synonymous mutations of dap2. Within the Australian clade of isolates, a cluster of isolates containing novel SpaA sequences 1-Q307R, 3 and 3-P323L were also observed to all exhibit frameshift mutations in the cbpA protein.

### 3.6. Resistance Gene Screening

All genomes were screened against the Resfinder database of antimicrobial resistance genes using Abricate. Of the 178 isolates examined in this study, 29 were identified by Abricate to contain at least one resistance gene. The isolates erysip_141, erysip_32, and erysip_91 were observed to contain three, two, and two resistance genes, respectively. Lincomycin resistance genes lsa(E), Lnu(B) and tetracycline resistance gene tet(M) were all observed in erysip_141 with identities of 96.77–99.9% with 100% coverage of the reference sequences. Lincomycin resistance was also observed in erysip_91 with lsa(E) and Lnu(B) having identities of 95.9% and 96.23% respectively with 100% coverage of the reference sequences. In addition, erysip_32 had 100% identity and coverage of a streptomycin resistance gene (str) and 87.17% identity and 98.57% coverage of the mph(B) erythromycin resistance gene, two genes not observed in any other Australian isolates. The remainder of the Australian isolates identified with a resistance gene all were observed to contain the tetracycline resistance gene tet(M) with 99.17–100% identity with 100% coverage of the reference sequences. Moreover, 29 isolates with resistance genes were identified by Ariba, 24 of which were observed to contain Oxytetracycline resistance. A complete table can be found in Supplementary Table S2. Of the 29 isolates identified to contain a resistance gene, 83% belonged to SpaA group 2-N101S (*n* = 22) or 2-N101S-I257L (*n* = 2). In addition, 79% (*n* = 23) belonged to ST 5 in the MLST scheme.

## 4. Discussion

In this study, Australian isolates of *E. rhusiopathiae* were investigated and compared to overseas isolates to determine the diversity and novelty of *E. rhusiopathiae* infections in Australian pig production. It is evident from the analyses presented here that the Australian isolates investigated comprise a large diversity of isolates and SpaA sequence types. In addition to the SpaA diversity, ten SpaA sequences were novel compared to previously reported sequences [9,18,20]. The diversity and novelty of these sequences is important to consider in the context of vaccine production and vaccine breakthrough in Australian swine production facilities. As previously reported, SpaA is an important protective antigen against *E. rhusiopathiae* and is commonly used as a candidate for vaccine production [18,43,44,45]. With worldwide recent outbreaks, investigations into the mechanisms of pathogenicity are uncovering potential links with SpaA types. Reported here are novel lineages of SpaA proteins, delineating a new group (Group 3) based on phylogenetic inference of the SpaA protein sequence.

In addition, 13 variants of SpaA groups 1 and 2 were also observed in Australian isolates. These novel lineages reported here displayed variant amino acids in the hypervariable region between positions 30 and 413 of the SpaA protein. Vaccine breakdown results in the dataset for novel SpaA sequences, while representing small sample numbers, shows the beginning of an interesting trend in which (excluding groups in <5 herds) previously reported SpaA sequence types such as groups 1, 2, 2-D195A and 2-I257L are associated with lower frequencies of observed vaccine breakdown in Australian piggeries than the novel SpaA types shown here in Australian isolates. A high percentage of vaccine breakdown was observed in isolates from SpaA groups 1-Q307R, 3 and 3-P323L (herd vaccine breakdowns of 35.3%, 42.86%, and 100%, respectively). Isolates displaying these SpaA variants were closely related when examined with the core gene phylogeny, all belonging to the same clade, except for 5 of 32 isolates with a group 1-Q307R sequence. Previous observations for *E. rhusiopathiae* phylogeny report a topology consisting of three clades and an intermediary clade, representing isolates containing SpaB, SpaA group 1, SpaA group 2, and the intermediate clade with a mixture of group 1 and 2 sequences [1]. Here, we also observe the same clustering of isolates with a group 1 SpaA sequence to clade 2 and group 2 SpaA sequences with clade 3, in line with previous studies [1,4,46,47]. However, isolates derived from Australian samples form a sub-clade within clade 3, generally associated with SpaA group 2 proteins. This sub-clade is comprised of multiple SpaA proteins, including group 1, 2, and 3 sequence types.

Discrepancy in the SpaA protein phylogeny versus the core gene phylogeny and the genomic relatedness of groups 1-Q307R, 3 and 3-P323L indicates that vaccine breakthrough is likely not due solely on the presence of novel SpaA types and that other genomics factors affecting vaccine efficacy are probable. The diversity of SpaA sequences in the Australian phylogenomic clade, in comparison to worldwide isolates being mostly limited to SpaA sequence types based on their presence in clades 2 and 3, suggests recombination may occur more frequently in Australian isolates to lead to this diversity. Correlation profiles exhibited monotonically decaying correlations indicating that recombination is present in *E. rhusiopathiae*, with these correlations previously observed in other recombining bacteria, such as Salmonella enterica, Klebsiella pneumoniae, Staphylococcus aureus and Pseudomonas Aeruginosa [41,48,49].

Vaccine isolates investigated in this study were observed to contain either a group 2-N101S or group 1 SpaA sequence type, relating to only 17.5% of isolates. While it is unknown whether any of these variants result in altered protection levels given by currently used vaccines, the higher vaccine breakdown rate observed here indicates that novel Australian isolates may be able to evade the protective benefits granted by vaccination. Australia also appears to have a relatively unique set of isolates in terms of sequence types based on MLST. Of 36 ST observed in isolates characterised from Australia, only five (14%) of these ST were observed in isolates from other geographic locations. In addition, multiple ST were observed for each SpaA and Serotype group. It is worth noting that Australia banned the import of pigs from all countries in response to the outbreak of African swine fever in 1959–1960 [50]. As such, the diversity of *E. rhusiopathiae* in Australia should not be influenced by worldwide trade of live animals. Worldwide trade restrictions likely play a role in the geographic separation of *E. rhusiopathiae*, observed in the topology of the core gene phylogeny. Screening genomes from Australian isolates with Abricate against the Resfinder database revealed streptomycin, tetracycline, and erythromycin resistance associated with 100% of isolates with ST 5. This ST represented 79.3% of all isolates identified by Abricate to possess resistance genes. Oxytetracycline resistance in *E. rhusiopathiae* was first reported in 1984 by Takahasi et al. [51] and has regularly been observed in swine isolates [7,46]. Extensive usage of tetracycline worldwide, especially in animal feed, is likely a contributing factor with previous observations of increased resistance determinants in pig isolates compared to wild boar isolates [46], likely reflecting industry antibiotic usage.

## 5. Conclusions

This study shows that Australia appears to have a unique diversity of *E. rhusiopathiae* isolates within pig production industries in addition to historical evidence of vaccine breakdown. The underlying biological structures, such as bacterial surface proteins, that are involved in eliciting protection of animals through vaccination, appear not be represented to the degree needed to provide appropriate protection against the large diversity of *E. rhusiopathiae* isolates in Australia. Australian isolates were observed to undergo higher rates of recombination in comparison to Clade 1 and Clade 2 isolates. *E. rhusiopathiae* is a major source of economic loss in the pork industry. Nationally this disease is not well understood due to the fact that most cases of erysipelas in Australia are dealt with on an on-farm basis. As such, working with historical data as that used in this study has potential limitations. Transfer of this knowledge between different individuals can result in incomplete datasets and inconsistent reporting of observations. While several samples’ metadata were annotated with information about vaccine breakdown, the vaccine status of all animals cannot be certain. This study highlights, however, the diversity of Australian isolates and opens the possibility for future work to confirm how this diversity may impact vaccination strategies. The results here describes the population structure and genomic features of 154 Australian *E. rhusiopathiae* isolates in the greater context of worldwide isolates, thus improving the knowledge base of *E. rhusiopathiae* in the Australian pig industry.

## Figures and Tables

**Table 1:** Vaccine breakdown among Australian *E. rhusiopathiae* cases.

Group	Vaccine Breakdown	Total	Breakdown (%)	Herds *	Breakdown Herd *	Breakdown Herd * (%)	Reference
1 **	7	35	17.14	25	5	20	Forde et al., 2020 [4]
2 **	1	4	25	4	1	25	Forde et al., 2020 [4]
3	5	9	55.56	7	3	42.86	This study
1-S124N-Q307R	1	1	100	1	1	100	This study
1-D195A-G303=	1	3	33.33	3	1	33.33	This study
1-Q307R	8	32	25	17	6	35.30	This study
1-Q307R-V400I	1	1	100	1	1	100	This study
1-K70 = -N101S	0	1	0	1	0	0	This study
2-N101S **	14	26	53.85	19	10	52.63	Janßen et al., 2015 [20]
2-N101S-I257L	2	2	100	1	1	100	This study
2-D195A **	3	42	7.14	20	3	15	Uchiyama et al., 2016 [41]
2-I257L **	0	5	0	4	0	0	Fujisawa reference sequence
2-I257L-M379I	0	1	0	1	0	0	This study
2-G54A-N101S	0	3	0	3	0	0	This study
3-P323L	6	6	100	1	1	100	This study

* Cases were grouped into herds when *E. rhusiopathiae* was collected on the same date at the same location. ** Previously identified SpaA groups.

## Data Availability

The data presented in this study are openly available in NCBI BioProject, accession number PRJNA909344.

## Supplementary Materials

The following supporting information can be downloaded at: https://www.mdpi.com/article/10.3390/microorganisms11020297/s1, Table S1: *E. rhusipathiae* metadata table, Table S2: Abricate results against Resfinder database.
