# Supplementary material for: Population Structure and Genomic Characteristics of Australian Erysipelothrix rhusiopathiae Reveals Unobserved Diversity in the Australian Pig Industry
Source: Microorganisms. 2023 Jan 23;11(2):297. doi: 10.3390/microorganisms11020297 (PMC9964597; doi:10.3390/microorganisms11020297)
Supplement: Supplementary file 1 [file microorganisms-11-00297-s001.zip › microorganisms-2114849-supplementary.pdf]

microorganisms

MDPI

Supplementary materials

Scheme 1. *E. rhusipathiae* metadata table.

	SpaA Group	Sequence Type	Source	Location	Year	Presentation	Amoxycillin (AML)	Erythromycin 'E'	Ceftiofur (EFT)	Lincospectin (LS)	Penicillin (P)	Oxytetracycline (OT)
erysip_1	1	7	Pig	Hungary	1981		-	-	-	-	-	-
erysip_2	1	103	Pig	NSW	1969	Acute	-	-	-	-	-	-
erysip_3	1	109			1981		-	-	-	-	-	-
erysip_4	1	85	Pig	NSW	1982	Sub-acute	-	-	-	-	-	-
erysip_5	2	81	Pig	NSW	1982	Acute	-	-	-	-	-	-
erysip_6	SpaB	108	Pig	USA	1982		-	-	-	-	-	-
erysip_7	SpaB	107		Argentina	1982		-	-	-	-	-	-
erysip_8	2-D195A	98	Pig	NSW	1983	Acute	-	-	-	-	-	-
erysip_9	2-D195A	77	Pig	NSW	1983	Chronic	-	-	-	-	-	-
erysip_10	2-I257L	94	Pig	NSW	1984		-	-	-	-	-	-
erysip_11	2-I257L	94	Pig	NSW	1984		-	-	-	-	-	-
erysip_12	1	85	Pig	NSW	1984	Acute	-	-	-	-	-	-
erysip_13	1	90	Pig	NSW	1986	Chronic	-	-	-	-	-	-
erysip_14	1	86	Pig		1987		-	-	-	-	-	-
erysip_15	2-I257L	88	Pig	NSW	1987	Acute	-	-	-	-	-	-
erysip_16	SpaB	106	Pig	NSW	1987	Sub-acute	-	-	-	-	-	-
erysip_17	1	86	Pig	NSW	1990	Acute	-	-	-	-	-	-
erysip_18	1	85	Pig	NSW	1994	Sub-acute	-	-	-	-	-	Resistant
erysip_19	2-N101S	5	Pig	NSW	1997	Acute	-	-	-	-	-	Resistant
erysip_20	1	76	Pig	NSW	1997	Acute	-	-	-	-	-	-
erysip_21	2-N101S	5	Pig	NSW	1998	Chronic	-	-	-	-	-	Resistant
erysip_22	2-N101S	5	Pig	NSW			-	-	-	-	-	Resistant
erysip_23	1	92	Vaccine	Vaccine			-	-	-	-	-	-
erysip_24	1	92	Vaccine	Vaccine			-	-	-	-	-	-
erysip_25	1-Q307R	102	Pig	WA	2000		-	-	-	-	-	-
erysip_26	2-N101S	5	Vaccine	Vaccine	2002		-	-	-	-	-	-

erysip_27	3	79	Pig	NSW	2008	Acute	-	-	-	-	-	-
erysip_28	3	75	Pig	NSW	2010	Acute	-	-	-	-	-	-
erysip_29	2-G54A-N101S	47	Pig	NSW	2010	Acute	-	-	-	-	-	Resistant
erysip_30	2-D195A	99	Pig		2010		-	-	-	-	-	-
erysip_31	2-G54A-N101S	47	Pig		2011		-	Resistant	-	-	-	Resistant
erysip_32	2-D195A	101	Pig	NSW	2011		-	-	-	-	-	-
erysip_33	2-G54A-N101S	47	Pig	NSW	2012	Chronic	-	-	-	-	-	Resistant
erysip_34	3	75	Pig	NSW	2013	Chronic	-	-	-	-	-	-
erysip_35	3	97	Pig	NSW	2016	Acute	-	-	-	-	-	-
erysip_36	2-D195A	48			1981		-	-	-	-	-	-
erysip_37	2	41			1981		-	-	-	-	-	-
erysip_38	1	95	Pig	NSW	1979	Acute	-	-	-	-	-	-
erysip_39	2-D195A	98	Pig	NSW	1981	Acute	-	-	-	-	-	-
erysip_40	2-I257L-M379I	87	Pig	NSW	1975	Acute	-	-	-	-	-	-
erysip_41	1	94	Pig	NSW	1982	Chronic	-	-	-	-	-	-
erysip_42	2-I257L	88	Pig	Hungary	1982		-	-	-	-	-	-
erysip_43	1	89	Pig	NSW	1982	Sub-acute	-	-	-	-	-	-
erysip_44	2-N101S	84	Pig	USA	1982		-	-	-	-	-	-
erysip_45	2-D195A	98	Pig	NSW	1983	Acute	-	-	-	-	-	-
erysip_46	1	90	Pig	NSW	1983	Acute	-	-	-	-	-	-
erysip_47	1	85	Pig	NSW			-	-	-	-	-	-
erysip_48	1	85	Pig	NSW			-	-	-	-	-	-
erysip_49	1	85	Pig	NSW	1983	Acute	-	-	-	-	-	-
erysip_50	2-D195A	76	Pig	NSW	1983	Chronic	-	-	-	-	-	-
erysip_51	1-K70=-N101S	94	Pig	NSW	1984	Acute	-	-	-	-	-	-
erysip_52	2-D195A	98	Pig	NSW	1985	Acute	-	-	-	-	-	-
erysip_53	1	9	Fish	Germany	1988		-	-	-	-	-	-
erysip_54	2	80	Pig	NSW	1988	Acute	-	-	-	-	-	-
erysip_55	1	94	Pig	NSW	1989	Chronic	-	-	-	-	-	-

erysip_56	1	73	Pig				-	-	-	-	-	-
erysip_57	1	73	Pig				-	-	-	-	-	-
erysip_58	1	73	Pig				-	-	-	-	-	-
erysip_59	2-D195A	99	Pig				-	-	-	-	-	-
erysip_60	2-D195A	99	Pig				-	-	-	-	-	-
erysip_61	2-N101S	5	Pig	NSW	1997	Acute	-	-	-	-	-	Resistant
erysip_62	2-N101S	5	Pig	NSW	1997		-	-	-	-	-	Resistant
erysip_63	2-N101S	5	Pig	NSW	1997	Sub-acute	-	-	-	-	-	Resistant
erysip_64	2-N101S	5	Pig	QLD	1997	Acute	-	-	-	-	-	Resistant
erysip_65	1-Q307R	76	Pig	NSW	1997	Acute	-	-	-	-	-	-
erysip_66	2-I257L	78	Pig	VIC	1997		-	-	-	-	-	-
erysip_67	2-D195A	99	Pig	WA	1997	Acute	-	-	-	-	-	-
erysip_68	1-Q307R	76	Pig	NSW	1997	Chronic	-	-	-	-	-	-
erysip_69	2-N101S	5	Pig	NSW	1997	Acute	-	-	-	-	-	Resistant
erysip_70	2-N101S	5	Pig	NSW	1997	Acute	-	-	-	-	-	Resistant
erysip_71	2-N101S	5	Pig	NSW	1998	Chronic	-	-	-	-	-	Resistant
erysip_72	2-N101S-I257L	5	Pig	NSW	1989	Chronic	-	-	-	-	-	Resistant
erysip_73	2-N101S-I257L	5	Pig	NSW	1989	Chronic	-	-	-	-	-	Resistant
erysip_74	2-N101S	5	Pig	NSW	1998	Chronic	-	-	-	-	-	Resistant
erysip_75	2-N101S	5	Pig	NSW	1998	Chronic	-	-	-	-	-	Resistant
erysip_76	2-D195A	80	Pig	NSW	1998	Chronic	-	-	-	-	-	-
erysip_77	1-D195A-G303=	98	Pig				-	-	-	-	-	-
erysip_78	1-D195A-G303=	98	Pig	NSW	1998	Sub-acute	-	-	-	-	-	-
erysip_79	1	96	Pig	QLD	1998	Chronic	-	-	-	-	-	-
erysip_80	2-N101S	5	Pig	NSW	1998	Sub-acute	-	-	-	-	-	Resistant
erysip_81	1-Q307R	76	Pig	NSW	1998	Chronic	-	-	-	-	-	-
erysip_82	1-Q307R-V400I	100	Pig	NSW	1998	Sub-acute	-	-	-	-	-	-
erysip_83	2-N101S	5	Pig	NSW	1998	Chronic	-	-	-	-	-	Resistant
erysip_84	2-N101S	5	Pig	VIC			-	-	-	-	-	Resistant

erysip_85	2-N101S	5	Pig	NSW	1998	Acute	-	-	-	-	-	Resistant
erysip_86	1-Q307R	90	Pig	NSW	1998	Chronic	-	-	-	-	-	-
erysip_87	3	75	Pig	NSW	1999	Chronic	-	-	-	-	-	-
erysip_88	1	92	Vaccine	Vaccine	1999		-	-	-	-	-	-
erysip_89	2-N101S	4	Pig	NSW	1999		-	-	-	-	-	Resistant
erysip_90	1-Q307R	76	Pig	NSW	1999	Chronic	-	-	-	-	-	-
erysip_91	1-Q307R	76	Pig	SA	1999		-	-	-	-	-	-
erysip_92	2-N101S	39	Pig	QLS	2000		-	-	-	-	-	Resistant
erysip_93	2-N101S	5	Vaccine	Vaccine	2000		-	-	-	-	-	Resistant
erysip_94	1-Q307R	76	Pig	NSW	2000	Chronic	-	-	-	-	-	-
erysip_95	1-Q307R	74	Pig	NSW	2000	Chronic	-	-	-	-	-	-
erysip_96	1-Q307R	76	Pig	NSW	2001		-	-	-	-	-	-
erysip_97	1-Q307R	76	Pig	VIC	2001		-	-	-	-	-	-
erysip_98	1-S124N-Q307R	104	Pig	QLS	2002		-	-	-	-	-	-
erysip_99	2-D195A	99	Pig	NSW	2002		-	-	-	-	-	-
erysip_100	1	85	Pig	NSW	2005	Acute	-	-	-	-	-	-
erysip_101	2-D195A	99	Pig	NSW	2006	Chronic	-	-	-	-	-	-
erysip_102	2-N101S	5	Pig	NSW	2006	Sub-acute	-	-	-	-	-	Resistant
erysip_103	1-Q307R	104	Pig	NSW	2007	Acute	-	-	-	-	-	-
erysip_104	1	94	Pig	NSW	1990	Sub-acute	-	-	-	-	-	-
erysip_105	1	92	Vaccine	Vaccine			-	-	-	-	-	-
erysip_106	2-D195A	90	Pig	NSW	2017	Acute	-	-	-	-	-	-
erysip_107	2-D195A	90	Pig	VIC	2017	Acute	-	-	Resistant	-	-	-
erysip_108		99		VIC	2017	Chronic	-	-	-	-	-	-
erysip_109		99		VIC	2017	Chronic	-	-	-	-	-	-
erysip_112	2-D195A	99	Pig	VIC	2017	Chronic	-	-	-	-	-	-
erysip_113		99		VIC	2017	Chronic	-	-	-	-	-	-
erysip_114	2-N101S	83	Pig	NSW	2017	Chronic	-	-	-	-	-	-
erysip_115	2-N101S	83	Pig	NSW	2017	Chronic	-	-	-	-	-	-
erysip_116	2-D195A	90	Pig	NSW	2017	Chronic	-	-	-	-	-	-
erysip_117	2-D195A	90	Pig	NSW	2017	Chronic	-	-	-	-	-	-
erysip_118	3-P323L	75	Pig	VIC	2017	Chronic	-	-	-	-	-	-
erysip_119	3-P323L	75	Pig	VIC	2017	Chronic	-	-	Resistant	-	-	-

erysip_120	3-P323L	75	Pig	VIC	2017	Chronic	-	-	-	-	-	-
erysip_121	SpaB	105	Pig	VIC	2017	Chronic	-	-	-	-	-	-
erysip_122	3-P323L	75	Pig	VIC	2017	Chronic	-	-	-	-	-	-
erysip_123	3-P323L	75	Pig	VIC	2017	Chronic	-	-	-	-	-	-
erysip_124	3-P323L	75	Pig	VIC	2017	Chronic	-	-	-	-	-	-
erysip_125	1-Q307R	82	Pig	VIC	2017	Chronic	-	-	-	-	-	-
erysip_126	1-Q307R	82	Pig	VIC	2017	Chronic	-	-	-	-	-	-
erysip_127	1	90	Pig	VIC	2017	Acute	-	-	-	-	-	-
erysip_128	1	91	Pig	VIC	2017	Acute	-	-	-	-	-	-
erysip_129	2-D195A	99	Pig	VIC	2017	Acute	-	-	-	-	-	-
erysip_130	2-D195A	93	Pig	VIC	2017		-	-	-	-	-	-
erysip_131	2-D195A	93	Pig	VIC	2017		-	-	-	-	-	-
erysip_132	2-D195A	99	Pig	VIC	2017		-	-	-	-	-	-
erysip_133	2-D195A	93	Pig	VIC	2017		-	-	-	-	-	-
erysip_134	2-D195A	93	Pig	VIC	2017		-	-	-	-	-	-
erysip_135	2-D195A	93	Pig	VIC	2017		-	-	-	-	-	-
erysip_136	2-D195A	93	Pig	VIC	2017		-	-	-	-	-	-
erysip_137	2-D195A	99	Pig	NSW	2016	Acute	-	-	-	-	-	-
erysip_138	3	75	Pig	NSW	2017		-	-	-	-	-	-
erysip_139	1-Q307R	76	Pig	NSW	2017		-	-	-	-	-	-
erysip_140	2-N101S	5	Pig	NSW	2017		-	-	-	-	-	Resistant
erysip_141	1	72	Pig	QLD	2017		-	-	-	-	-	-
erysip_142	1-Q307R	76	Pig	NSW	2017		-	-	-	-	-	-
erysip_143	2	80	Pig	NSW	2017		-	-	-	-	-	-
erysip_144	1-D195A-G303=	98	Pig	NSW	2017		-	-	-	-	-	-
erysip_145	3	75	Pig	NSW	2017		-	-	-	-	-	-
erysip_146	3	75	Pig	NSW	2017		-	-	-	-	-	-
erysip_147	2-N101S	5	Pig	NSW	2017		-	-	-	-	-	Resistant
erysip_148	1-Q307R	76	Pig	NSW	2017		-	-	-	-	-	-
erysip_149	1-Q307R	90	Pig	NSW	2017		-	-	-	-	-	-
erysip_150	1-Q307R	76	Pig	NSW	2017		-	-	-	-	-	-
erysip_151	1-Q307R	76	Pig	QLD	2017		-	-	-	-	-	-
erysip_152	1-Q307R	74	Pig	QLD	2017		-	-	-	-	-	-

erysip_153	1-Q307R	76	Pig	NSW	2017	Chronic	-	-	-	-	-	-
erysip_154	1-Q307R	76	Pig	NSW	2017	Chronic	-	-	-	-	-	-
erysip_155	1-Q307R	76	Pig	NSW	2017	Chronic	-	-	-	-	-	-
erysip_156	1-Q307R	76	Pig	NSW	2017	Chronic	-	-	-	-	-	-
erysip_157	1-Q307R	76	Pig	NSW	2017	Chronic	-	-	-	-	-	-
erysip_158	1-Q307R	76	Pig	NSW	2017	Chronic	-	-	-	-	-	-
erysip_159	3	97	Pig	NSW	2017	Acute	-	-	-	-	-	-
erysip_160	1-Q307R	76	Pig	NSW	2017	Sub-acute	-	-	-	-	-	-
erysip_161	2-D195A	99	Pig	VIC	2017		-	-	Resistant	-	Resistant	-
erysip_162	2-D195A	99	Pig	VIC	2017		-	-	Resistant	Resistant	-	-
erysip_163	2-D195A	99	Pig	VIC	2017		-	-	-	-	-	-
erysip_164	2-D195A	99	Pig	VIC	2017		-	-	-	-	-	-
erysip_165	2-D195A	99	Pig	VIC	2017		-	-	-	-	-	-
erysip_166	1-Q307R	76	Pig	NSW	2017	Chronic	-	-	-	-	-	-
erysip_167	1-Q307R	76	Pig	NSW	2017	Chronic	-	-	-	-	-	-
erysip_168	1-Q307R	76	Pig	NSW	2017	Chronic	-	-	-	-	-	-
erysip_169	2-D195A	99	Pig	VIC	2017	Acute	-	-	-	-	-	-
erysip_170	2-D195A	93	Pig	VIC	2017		-	-	-	-	-	-
erysip_171	2-D195A	93	Pig	VIC	2017		-	-	-	-	-	-
erysip_172	2-D195A	93	Pig	VIC	2017		-	-	-	-	-	-
erysip_173	2-D195A	93	Pig	VIC	2017		-	-	-	-	-	-
erysip_174	2-D195A	99	Pig	VIC	2017		-	-	-	-	-	-
erysip_175	1-Q307R	76	Pig	NSW	2017	Acute	-	-	-	-	-	-
erysip_176	2-D195A	80	Pig	NSW	2017		-	-	-	-	-	-
erysip_177	1	90	Pig	VIC	2017	Acute	-	-	-	-	-	-
erysip_178	1	90	Pig	VIC	2017	Acute	-	-	-	-	-	-
erysip_179	1	90	Pig	VIC	2017	Acute	-	-	-	-	-	-
erysip_180	2-D195A	93	Pig	VIC	2017		-	-	-	-	-	-

Scheme 2. Abricate results against Resfinder database.

Isolate	Gene	Coverage	Gaps	Percent Coverage	Percent Identity	Accession	SpaA Group	ST Group	Oxytetracycline resistance
erysip_4	tet(M)_1	1-1920/1920	0/0	100	100	X92947	1	85	S
erysip_18	tet(M)_1	1-1920/1920	0/0	100	100	X92947	1	85	Resistant
erysip_19	tet(M)_4	1-1920/1920	0/0	100	99.17	X75073	2-N101S	5	Resistant
erysip_21	tet(M)_4	1-1920/1920	0/0	100	99.17	X75073	2-N101S	5	Resistant
erysip_22	tet(M)_4	1-1920/1920	0/0	100	99.17	X75073	2-N101S	5	Resistant
erysip_26	tet(M)_4	1-1920/1920	0/0	100	99.17	X75073	2-N101S	5	S
erysip_32	str_1	1-849/849	0/0	100	100	X92946	2-D195A	101	S
erysip_32	mph(B)_1	1-896/909	0/0	98.57	87.17	D85892	2-D195A	101	S
erysip_61	tet(M)_4	1-1920/1920	0/0	100	99.17	X75073	2-N101S	5	Resistant
erysip_62	tet(M)_4	1-1920/1920	0/0	100	99.17	X75073	2-N101S	5	Resistant
erysip_63	tet(M)_4	1-1920/1920	0/0	100	99.17	X75073	2-N101S	5	Resistant
erysip_64	tet(M)_4	1-1920/1920	0/0	100	99.17	X75073	2-N101S	5	Resistant
erysip_69	tet(M)_4	1-1920/1920	0/0	100	99.17	X75073	2-N101S	5	Resistant
erysip_70	tet(M)_4	1-1920/1920	0/0	100	99.17	X75073	2-N101S	5	Resistant
erysip_71	tet(M)_4	1-1920/1920	0/0	100	99.17	X75073	2-N101S	5	Resistant
erysip_72	tet(M)_4	1-1920/1920	0/0	100	99.17	X75073	2-N101S-I257L	5	Resistant

erysip_73	tet(M)_4	1-1920/1920	0/0	100	99.17	X75073	2-N101S-I257L	5	Resistant
erysip_74	tet(M)_4	1-1920/1920	0/0	100	99.17	X75073	2-N101S	5	Resistant
erysip_75	tet(M)_4	1-1920/1920	0/0	100	99.17	X75073	2-N101S	5	Resistant
erysip_80	tet(M)_4	1-1920/1920	0/0	100	99.17	X75073	2-N101S	5	Resistant
erysip_83	tet(M)_4	1-1920/1920	0/0	100	99.17	X75073	2-N101S	5	Resistant
erysip_84	tet(M)_4	1-1920/1920	0/0	100	99.17	X75073	2-N101S	5	Resistant
erysip_85	tet(M)_4	1-1920/1920	0/0	100	99.17	X75073	2-N101S	5	Resistant
erysip_89	tet(M)_4	1-1920/1920	0/0	100	99.17	X75073	2-N101S	4	Resistant
erysip_91	lsa(E)_1	1-1485/1485	0/0	100	96.23	JX560992	1-Q307R	76	S
erysip_91	lnu(B)_2	1-804/804	0/0	100	95.9	JQ861959	1-Q307R	76	S
erysip_93	tet(M)_4	1-1920/1920	0/0	100	99.17	X75073	2-N101S	5	Resistant
erysip_102	tet(M)_4	1-1920/1920	0/0	100	99.17	X75073	2-N101S	5	Resistant
erysip_140	tet(M)_4	1-1920/1920	0/0	100	99.17	X75073	2-N101S	5	Resistant
erysip_141	tet(M)_4	1-1920/1920	0/0	100	99.9	X75073	1	72	S
erysip_141	lnu(B)_2	1-804/804	0/0	100	96.89	JQ861959	1	72	S
erysip_141	lsa(E)_1	1-1485/1485	0/0	100	96.77	JX560992	1	72	S
erysip_147	tet(M)_4	1-1920/1920	0/0	100	99.17	X75073	2-N101S	5	Resistant
